# Comments on: Randomized clinical trials and the proportional hazards model for recurrent events

**DOI:** 10.1007/s11749-026-01027-6

**Published:** 2026-06-29

**Authors:** Robert L. Strawderman, Ashkan Ertefaie

**Affiliations:** 1 Department of Biostatistics and Computational Biology, University of Rochester School of Medicine and Dentistry, 265 Crittenden Boulevard, Rochester, New York 14642 USA; 2 Department of Biostatistics, Epidemiology, and Informatics, University of Pennsylvania Perelman School of Medicine, 423 Guardian Dr, Philadelphia, Pennsylvania 19104 USA

We thank the editor for the invitation to comment on this interesting paper. Our comments will initially focus on the authors’ derivation of the influence function ϕ for estimating $\beta ^*;$ we will then explore its connections with both $U^{ipcw,pl}(\beta )$ and $U^{aipcw,pl}(\beta )$ and propose a class of weighted estimating functions for estimating $\beta ^*$ related to the latter.

Let $Z^F(s,A) = \int _0^s (A - e(u)) G_c(u,A) d M^F(u,A)$ where, unless otherwise noted, we make use of the authors’ notation and assumptions. We begin by noting that ϕ can be rewritten as below:

$$\begin{aligned} \phi = Z^F(\tau ,A) - \int _0^\tau \left\{ Z^F(\tau ,A) - Z^F(s,A) \right\} \frac{d M^C(s,A)}{G_c(s,A)}. \end{aligned}$$

While explicit details are not provided, the paper narrative suggests that ϕ is derived directly from the (unscaled) observed data influence function $\psi = \int _0^\tau (A - e(t) ) d M(t,A)$ for estimating $\beta ^*$ via the “usual partial likelihood score of the observed data.” Specifically, noting $d M(t,A) = Y(t) d M^F(t,A)$ for $Y(t) = I\{ T \ge t\}$ and $d M^F(t,A)= I\{D \ge t\} [d N^*(t) - e^{\beta ^* \! A} \lambda _0(t) dt],$ we can first write

1 $$\begin{aligned} \psi= &  \int _0^\tau (A - e(t) ) G_c(t,A) \frac{Y(t)}{G_c(t,A)} d M^F(t,A); \end{aligned}$$

then, upon substituting into (1) the “identity”

2 $$\begin{aligned} \frac{Y(t)}{G_c(t,A)} = 1 - \int _0^t \frac{d M^C(s,A)}{G_c(s,A)}, \end{aligned}$$

simplification leads to the stated expression for ϕ.

The term “identity” is in quotes because (2) does not hold as stated. Rather, using results in Baer et al. (2025, Lemma 2), the following modified identity holds:

3 $$\begin{aligned} \frac{Y(t)}{G_c(t,A)} = Y(t) \left\{ 1 - \int _0^t \frac{d M^C(s,A)}{G_c(s,A)} \right\} . \end{aligned}$$

Substituting (3) into (1), defining $Z^O(s,A) = \int _0^s (A - e(u)) G_c(u,A) I\{ C \ge u \} d M^F(u,A),$ and simplifying, we are instead led to

$$\begin{aligned} \psi = Z^O(\tau ,A) - \int _0^\tau \left\{ Z^O(\tau ,A) - Z^O(s,A) \right\} \frac{d M^C(s,A)}{G_c(s,A)}. \end{aligned}$$

Because $Z^O(s,A) = Z^F(s \wedge T,A)$ for any $s \in (0,\tau ],$ we see that ψ generally differs from ϕ and does not decompose ψ into a complete data score plus an orthogonal censoring term.

An immediate question, then, is how might ϕ arise. Define for any $t \le \tau$ and a suitable known function *k*(*u*, *A*) the full data quantity

4 $$\begin{aligned} \psi ^F(\beta ^*,t; k)=\int _0^{t} \left\{ A - {{\mathcal {E}}}(u,\beta ^*;k) \right\} k(u,A) \, dM^F(u,A), \end{aligned}$$

where

$$\begin{aligned} {{\mathcal {E}}}(u,\beta ;k) = \frac{ E[ A I\{D \ge u\} k(u,A) e^{\beta A}]}{E[ I\{D \ge u\} k(u,A) e^{\beta A}]}; \end{aligned}$$

note that $k(u,A) = G_c(u,A)$ gives ${{\mathcal {E}}}(u,\beta ^*;G_c) = e(u),$ and $\psi ^F(\beta ^*,\tau ; G_c) = H^F(0,A)$ as defined in the paper. Clearly, $\psi ^F(\beta ^*,t;k)$ depends only on the full data $(D,{\bar{N}}^*(D),A).$ Letting $\mathcal {H}(u) = \{ A, {\bar{N}}^*(u) \}$ and following Tsiatis (2006, Sec. 9.3), the class of all observed data influence functions for estimating $\beta ^*$ based on (4) under the censoring assumptions of this article is given by

5 $$\begin{aligned} \frac{\Delta }{G_c(T,A)} \psi ^F(\beta ^*,\tau ;k) + \int _0^\infty b(s;D, \mathcal {H}(s)) \frac{d M^C(s,A)}{G_c(s,A)}, \end{aligned}$$

where *b* denotes a suitable function of the indicated data. Using the well-known identity

6 $$\begin{aligned} \frac{\Delta }{G_c(T,A)} = 1 - \int _0^{\infty } \frac{d M^C(s,A)}{G_c(s,A)}, \end{aligned}$$

substitution into (5) and simplification give the equivalent representation

7 $$\begin{aligned} \psi ^F(\beta ^*,\tau ;k) - \int _0^{\infty } \left\{ \psi ^F(\beta ^*,\tau ;k) - b(s; D, \mathcal {H}(s)) \right\} \frac{d M^C(s,A)}{G_c(s,A)}. \end{aligned}$$

Setting $b(s; D, \mathcal {H}(s)) = \psi ^F(\beta ^*,s;k)$ and recognizing that $k(u,A) = G_c(u,A)$ leads to $\psi ^F(\beta ^*,t;G_c) - \psi ^F(\beta ^*,s;G_c) = H^F(s,A),$ we now recover ϕ from (7).

The above argument suggests that ϕ corresponds to the (unscaled) influence function of the solution to $U^O_{n,ipcw}(\beta , \tau ) = 0,$ where

$$\begin{aligned}&U^O_{n,ipcw}(\beta , t) = \\&\hspace{5mm} \mathbb {P}_n \frac{\Delta }{G_c(T,A)} \int _0^{t} \left\{ A - \frac{\mathbb {P}_n \frac{\Delta }{G_c(T,A)} G_c(u,A) A e^{\beta A} I\{ D \ge u\} }{\mathbb {P}_n \frac{\Delta }{G_c(T,A)} G_c(u,A) e^{\beta A} I\{ D \ge u\} } \right\} G_c(u,A) I\{ D \ge u\} d N^*(u). \end{aligned}$$

The estimating equations $U^O_{n,ipcw}(\beta , \tau )$ and $U^{ipcw,pl}(\beta )$ are different; indeed, it is reasonable to expect the latter to be more efficient since it uses, in effect, a time-dependent IPCW weight. This calls into question both the form of the ϕ-based variance estimator for the solution to $U^{ipcw,pl}(\beta )$ and the stated motivation for the “best censoring augmented estimator” that solves $U^{aipcw,pl}(\beta ).$

Below, we consider an alternative approach to improving upon $U^{ipcw,pl}(\beta )$ as a basis for estimating $\beta ^*.$ Define

$$\begin{aligned} U^O_n(\beta , t;w)= \mathbb {P}_n \int _0^{t} w(u,A) \left\{ A - \frac{\mathbb {P}_n w(u,A) \frac{Y(u)}{G_c(u,A)} A e^{\beta A}}{\mathbb {P}_n w(u,A) \frac{Y(u)}{G_c(u,A)} e^{\beta A}} \right\} \, \frac{Y(u)}{G_c(u,A)} d N(u); \end{aligned}$$

solving $U^O_n(\beta , \tau ;w) = 0$ leads to $\hat{\beta }_{n,w},$ say. Defining $d M(t,A;\beta ) = Y(t) d M^F(t,A;\beta )$ for $d M^F(t,A;\beta ) = I\{D \ge t\} [d N^*(t) - e^{\beta \! A} \lambda _0(t) dt],$ a familiar calculation shows that

$$\begin{aligned} U^O_n(\beta ;w) = \mathbb {P}_n \int _0^{\tau } w(u,A) \left\{ A - \frac{\mathbb {P}_n w(u,A) \frac{Y(u)}{G_c(u,A)} A e^{\beta A}}{\mathbb {P}_n w(u,A) \frac{Y(u)}{G_c(u,A)} e^{\beta A}} \right\} \, \frac{Y(u)}{G_c(u,A)} d M^F(u,A;\beta ). \end{aligned}$$

Under typical regularity conditions,

$$\begin{aligned} \sup _{u \in [0,\tau ]} \left\| \frac{\mathbb {P}_n w(u,A) \frac{Y(u)}{G_c(u,A)} A e^{\beta A}}{\mathbb {P}_n w(u,A) \frac{Y(u)}{G_c(u,A)} e^{\beta A}} - {{\mathcal {E}}}(u,\beta ;w) \right\| {\mathop {\rightarrow }\limits ^{p}} 0; \end{aligned}$$

hence, as an estimator for $\beta ^*,$ $\hat{\beta }_{n,w}$ should have influence function

$$\begin{aligned} \psi ^O(\beta ^*;w) = \int _0^{\tau } w(u,A) \left\{ A - {{\mathcal {E}}}(u,\beta ^*; w) \right\} \, \frac{Y(u)}{G_c(u,A)} d M^F(u,A). \end{aligned}$$

Under the authors’ censoring assumptions, we have $E[\psi ^O(\beta ^*;w) | A,D,{\bar{N}}(D)] = \psi ^F(\beta ^*,\tau ;w),$ where $\psi ^F(\beta ^*,\tau ;w)$ is defined in (4). Proceeding similarly to van der Laan and Robins (2003, Sec. 3.3.2) (see also Miloslavsky et al. (2004) for an application of these results to a very similar problem), the influence function that corresponds the “optimal” (i.e., variance minimizing) choice of observed data estimating function *that can be derived by augmenting*  $U^O_n(\beta ;w)$ is given by $\psi ^{opt}(\beta ^*;w) = \psi ^O(\beta ^*;w) - \int _0^{\infty } Q(u,\mathcal {F}(u);w)\,dM^C(u,A)$ where ${{\mathcal {F}}}(u) = \{ {{\mathcal {H}}}(u), D \ge u\}$ and

$$\begin{aligned} Q(u,\mathcal {F}(u);w)&= E[ \psi ^O(\beta ^*;w) \mid \mathcal {F}(u), C=u] -E[ \psi ^O(\beta ^*;w) \mid \mathcal {F}(u), C> u] \\&= E\left[ \int _u^{\tau } w(s,A) \left\{ A - {{\mathcal {E}}}(s,\beta ^*;w) \right\} \, \frac{Y(s)}{G_c(s,A)} dM^F(s,A) \Bigm | \mathcal {F}(u), C > u \right] \\&= \frac{1}{G_c(u,A)} E\left[ \psi ^F(\beta ^*,\tau ;w) - \psi ^F(\beta ^*,u;w) \mid \mathcal {H}(u), D \ge u \right] , \end{aligned}$$

the last step following from the definition of $\psi ^F(\beta ^*,t;w)$ in (4). A brief proof can be found in the appendix. In particular, it follows that

$$\begin{aligned} \psi ^{opt}(\beta ^*,\tau ;w) = \psi ^O(\beta ^*,\tau ;w) - \int _0^{\infty } E\left[ \psi ^F(\beta ^*,\tau ;w) - \psi ^F(\beta ^*,u;w) \mid \mathcal {H}(u), D \ge u \right] \frac{d M^C(u,A)}{G_c(u,A)}. \end{aligned}$$

Suppose we again select $w(u,A) = G_c(u,A),$ implying $\psi ^F(\beta ^*,\tau ;G_c) = H^F(0,A)$ and $\psi ^F(\beta ^*,\tau ;w) - \psi ^F(\beta ^*,u;w) = H^F(u,A);$ this results in the influence function

$$\begin{aligned} \psi ^{opt}(\beta ^*,\tau ;G_c) = \psi ^O(\beta ^*,\tau ;G_c) - \int _0^{\infty } E\left[ H^F(u,A) \mid \mathcal {H}(u), D \ge u \right] \frac{d M^C(u,A)}{G_c(u,A)}. \end{aligned}$$

In view of the fact that $U^O_n(\beta ;G_c) = U^{ipcw,pl}(\beta )$ and considering the relationship between $U^O_n(\beta ;G_c)$ and $\psi ^O(\beta ^*,\tau ;G_c),$ this argument clearly justifies $U^{aipcw,pl}(\beta )$ as the best improvement (i.e., largest variance reduction) over $U^{ipcw,pl}(\beta ).$ For such improvement to occur, $U^{ipcw,pl}(\beta )$ must be negatively correlated with the augmentation term; orthogonality would instead inflate the variance. These same statements apply to using an augmented version of $U^O_n(\beta ;w)$ *for any given choice of weight function*. Importantly, selecting *w*(*u*, *A*) to be something other than $G_c(u,A)$ may lead to an estimator of $\beta ^*$ with smaller variance. Consider, for example, w(u,A)=1. Then,

$$\begin{aligned} \psi ^{opt}(\beta ^*,\tau ;1) = \psi ^O(\beta ^*,\tau ;1) - \int _0^{\infty } E\left[ \psi ^F(\beta ^*,\tau ;1) - \psi ^F(\beta ^*,u;1) \mid \mathcal {H}(u), D \ge u \right] \frac{d M^C(u,A)}{G_c(u,A)}. \end{aligned}$$

Here, $\psi ^F(\beta ^*,u;1) = \int _0^u (A - {{\mathcal {E}}}(s,\beta ^*;1)) d M^F(s,A),$ which, unlike $\psi ^F(\beta ^*,u;G_c),$ does not depend on the censoring mechanism. This is arguably a more natural starting point for developing an estimating equation for $\beta ^*$ in the absence of censoring. Whether it has smaller variance than that derived from $U^O_n(\beta ;G_c)$ is unclear. It would be interesting to study how the choice of weight function impacts efficiency in this problem.

## Data Availability

Data availability and sharing is not applicable; no new data were generated, collected, or analyzed.

## Appendix

Detailed argument for establishing the form of $Q(u,\mathcal {F}(u);w),$ assuming $G_c(u,A)$ is a continuous function for $u \in [0,\tau ]$:

$$\begin{aligned} Q(u,\mathcal {F}(u);w)&= E[ \psi ^O(\beta ^*;w) \mid \mathcal {F}(u), C=u] -E[ \psi ^O(\beta ^*;w) \mid \mathcal {F}(u), C> u] \\&= E\left[ \int _u^{\tau } w(s,A) \left\{ A - {{\mathcal {E}}}(s,\beta ^*;w) \right\} \, \frac{Y(s)}{G_c(s,A)} dM^F(s,A) \Bigm | \mathcal {F}(u), C> u \right] \\&= \frac{E\left[ I\{C> u\} \int _u^{\tau } w(s,A) \left\{ A - {{\mathcal {E}}}(s,\beta ^*;w) \right\} \, \frac{I\{C \ge s\} }{G_c(s,A)} I\{D \ge s\} [d N^*(s) - e^{\beta \! A} \lambda _0(s) ds] \bigm |\mathcal {F}(u) \right] }{P\{ C > u | \mathcal {F}(u) \}} \\&= \frac{1}{G_c(u,A)} \int _u^{\tau } E\left[ w(s,A) \left\{ A - {{\mathcal {E}}}(s,\beta ^*;w) \right\} \, \frac{I\{C \ge s\} }{G_c(s,A)} [d N^*(s) - e^{\beta \! A} \lambda _0(s) ds] \Bigm | \mathcal {F}(u) \right] \\&= \frac{1}{G_c(u,A)} \int _u^{\tau } E\left[ E\left[ w(s,A) \left\{ A - {{\mathcal {E}}}(s,\beta ^*;w) \right\} \, \frac{I\{C \ge s\} }{G_c(s,A)} [d N^*(s) - e^{\beta \! A} \lambda _0(s) ds] \Bigm | \mathcal {F}(s) \right] \Bigm | \mathcal {F}(u)\right] \\&= \frac{1}{G_c(u,A)} \int _u^{\tau } E\left[ w(s,A) \left\{ A - {{\mathcal {E}}}(s,\beta ^*;w) \right\} \, [d N^*(s) - e^{\beta \! A} \lambda _0(s) ds] \Bigm | \mathcal {F}(u)\right] \\&= \frac{1}{G_c(u,A)} E\left[ \int _u^{\tau } w(s,A) \left\{ A - {{\mathcal {E}}}(s,\beta ^*;w) \right\} \, dM^F(s,A) \mid \mathcal {F}(u) \right] \\&= \frac{1}{G_c(u,A)} E\left[ \int _u^{\tau } w(s,A) \left\{ A - {{\mathcal {E}}}(s,\beta ^*;w) \right\} \, dM^F(s,A) \mid \mathcal {H}(u), D \ge u \right] \\&= \frac{1}{G_c(u,A)} E\left[ \psi ^F(\beta ^*,\tau ;w) - \psi ^F(\beta ^*,u;w) \mid \mathcal {H}(u), D \ge u \right] , \end{aligned}$$
